# Interplanting Annual Ryegrass, Wheat, Oat, and Corn to Mitigate Iron Deficiency in Dry Beans

**DOI:** 10.1371/journal.pone.0115673

**Published:** 2014-12-23

**Authors:** Emmanuel Chiwo Omondi, Andrew R. Kniss

**Affiliations:** 1 Department of Ecosystem Science and Management, University of Wyoming, 1000 East University Avenue, Laramie, Wyoming, 82071, United States of America; 2 Department of Plant Sciences, University of Wyoming, 1000 East University Avenue, Laramie, Wyoming, 82071, United States of America; Department of Agriculture and Food Western Australia, Australia

## Abstract

This study evaluated whether grass intercropping can be used to alleviate Fe deficiency chlorosis in dry beans (*Phaseolus vulgaris* L.) grown in high pH, calcareous soils with low organic matter. Field studies were conducted at the University of Wyoming Sustainable Agriculture Research and Extension Center in 2009 and 2010. Black- and navy beans were grown alone or intercropped with annual ryegrass (*Lolium multiflorum* Lam.), oat (*Avena sativa* L.), corn (*Zea mays* L.), or spring wheat (*Triticum aestivum* L.) in a two-factor factorial strip-plot randomized complete block design. All four grass species increased chlorophyll intensity in dry beans. However, grass species did not increase iron (Fe) concentration in dry bean tissues suggesting inefficient utilization of Fe present in the dry bean tissues. In 2009, nitrate-nitrogen (NO_3_-N) and manganese (Mn) concentration in bean tissue were greater in bean monoculture than in grass intercropped beans. Bean monoculture also had greater soil NO_3_-N concentrations than grass intercropped treatments. In 2009, grass intercrops reduced dry bean yield >25% compared to bean monoculture. Annual ryegrass was the least competitive of the four annual grass species. This suggests that competition from grasses for nutrients, water, or light may have outweighed benefits accruing from grass intercropping. Additional studies are required to determine the appropriate grass and dry bean densities, as well as the optimum time of grass removal.

## Introduction

The northern Great Plains is a key contributor to dry edible bean production in the United States accounting for 52% of the total dry bean production in 2008 [1]. Iron deficiency chlorosis in dry bean is common in the high pH, calcareous soils prevalent in this region [2]. Iron deficiency chlorosis under these conditions may not be due to low Fe concentration in soil but more frequently a result of low Fe availability [3]. Iron is almost exclusively present in the soil in its oxidized form, Fe (III), whose availability for plant uptake is limited due to its low solubility, especially at high pH and in the presence of oxygen [4], [5], [6]. In calcareous soils, constituting over one third of global cultivated land [7], the soil solution provides less than one tenth of plants' requirement for Fe [8]. Iron is essential for many physiological and biochemical processes that drive the biotic system including photosynthesis, respiration, DNA synthesis and dinitrogen fixation [9].

Conventional management of Fe deficiency involves foliar application of 1 to 2% of Fe sulfate solution (FeSO4.7H2O) at 200 to 400 l ha^−1^) [2], [10]. An understanding of cultural options of managing Fe deficiency is important in determining more sustainable and less expensive alternatives for conventional, organic, and natural dry bean producers. One such option may involve utilizing synergistic relationships that may exist between plant species that are tolerant and susceptible to Fe deficiency grown together in polycultures.

Higher plants have developed two different Fe-efficient strategies to increase Fe availability in soils; referred to as Strategy I and II [11]. Strategy I, found in dicot and non-graminaceous monocot species in response to Fe deficiency, involves acidification of the rhizosphere by the plants through proton (H^+^) extrusion, increasing Fe^3+^-chelates' solubility and the concomitant reduction by a ferric reductase to Fe^2+^, which can then be taken up by the plants [12]. In Strategy II plants found in Poaceae species, such as wheat (*Triticum aestivum* L.), barley (*Hordeum vulgare* L.), and corn (*Zea mays* L.), phytosiderophores are synthesized by the plant roots and secreted in the rhizosphere where they chelate Fe^3+^ and make it more bioavailable [13], [6], [14]. In high pH calcareous soils with elevated bicarbonate content, the activity of Strategy I can be neutralized, causing Fe deficiency chlorosis in the plants [10]. Venkat and Marschner [15] showed that release of reducing substances by Fe-efficient Strategy I plants under Fe deficiency declined at high pH level during the growth of sunflower (*Helianthus annuus* L.) in nutrient solution.

As with most micronutrients, the concentration of Fe in the soil can be altered by the availability of other nutrients creating antagonistic or synergistic effects [16]. For example, Ambler et al. [17] found that soil Zn interfered with the translocation of Fe in soybean by inhibiting the capacity of the root conversion of ferric to ferrous iron or by accentuating other reactions detrimental to Fe transport. Aktas and Van Egmond [18], Mengel [3], Mengel et al. [19], and Bloom et al. [20] have shown that high nitrate-nitrogen (NO_3_-N) concentration in the soil can also induce Fe chlorosis. Bloom et al. [20] attributed the greater chlorophyll intensity in soybean [*Glycine max* (L.)] plants growing on wheel tracks to lower soil and leaf tissue NO_3_-N on those tracks compared to adjacent areas that had more chlorotic soybeans. They also found significantly greater soil and leaf tissue NO_3_-N of the more chlorotic soybean monoculture than the less chlorotic soybean plants intercropped with oat. Bloom et al. [20] also demonstrated that increasing soil NO_3_-N through fertilization resulted in increased Fe deficiency chlorosis and decreased yields of the Fe-deficiency tolerant soybean variety used in the study. In addition to the proton extrusion strategy developed by Strategy I plants in response to Fe stress, N nutritional status considerably influences proton or hydroxyl (OH-) ion excretion from plant roots [18]. Plant species growing in complete nutrient solution with NO_3_-N exude OH- or HCO- into the nutrient medium as long as there is sufficient NO_3_ in the medium [21], [18]. Iron efficient plants secrete H^+^ ions into the rhizosphere when Fe stress develops, regardless of the NO_3_ status of the soil, and continue to excrete protons even after soil NO_3_ supply is depleted [18]. Iron inefficient plants, on the other hand, continue to secrete OH^−^ and HCO^−^ ions into the rhizosphere when NO_3_ is sufficiently available, even when Fe stress develops, with excretion of protons beginning only after NO_3_ supply in the soil has been depleted. Elevated HCO_3_^−^ concentration can make Fe insoluble thereby inhibiting its uptake by roots and subsequent translocation into shoots and leaves [18], [22], [23], [24]. Kosegarten et al. [24] found that NO_3_^−^ solution culture and simulated calcareous soil solution (NO_3_^−^ together with bicarbonate) severely affected the physiological Fe efficiency in roots and leaves of sunflower resulting in Fe deficiency chlorosis symptoms.

Practiced worldwide for many generations, mixed cropping, especially of legumes and grasses, can enhance on-farm biodiversity, promote biological N fixation, increase dry matter production and grain yield, and enhance resource use efficiency [25], [26], [27], [28], [29]. Plant cultivars that are tolerant to Fe deficiency selectively intercropped with susceptible plant cultivars can alleviate Fe chlorosis of the latter. In comparative studies between Fe-efficient sunflower plant species and Fe-inefficient corn species, Venkat Raju and Marschner [15] and Kashirad and Marschner [21] showed that under Fe deficiency conditions, sunflower plants lowered the pH of the nutrient solution resulting in increased uptake of inorganic Fe^3+^ evidenced by re-greening of the sunflower plants. Contrary to this observation, corn plants were not able to lower the pH of the nutrient solution as a result of which they were unable to utilize Fe^3+^ as a source of Fe. When the two plant species were intercropped in the nutrient solution under Fe-stress, the Fe-efficient sunflower lowered the pH of the nutrient solution enabling corn plants to also re-green with Fe^3+^ as the source of Fe.

In 2002, a Wyoming farmer observed that pinto beans accidentally growing with annual ryegrass were less chlorotic and produced better yields than a nearby monoculture of pinto beans growing in the absence of ryegrass. A subsequent bean-ryegrass intercropping on-farm study by Omondi et al. [30] revealed that annual ryegrass increased soil Fe concentration, extracted using the diethylenetriaminepentaacetic acid (DTPA) [42], by 23% when intercropped with pinto beans. Whereas the potential of annual ryegrass to alleviate Fe deficiency chlorosis was demonstrated, the increase in soil Fe concentration was only marginal, and pinto bean yields were not improved by ryegrass. The objective of this study was to determine whether interplantings of four annual grass species (annual ryegrass, oat, corn, and wheat), with dry bean can mitigate Fe deficiency in calcareous soils.

## Materials and Methods

**Figure 1:**
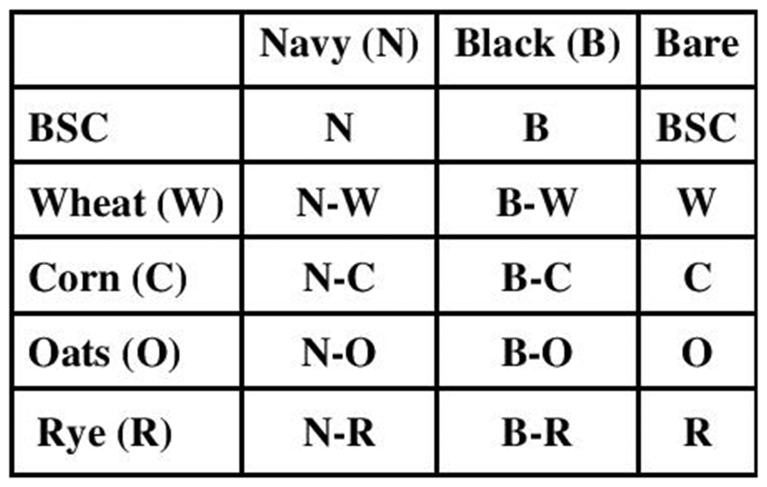
Experimental plot plan for one of the four replicates of the study showing rows of grass species (sub-plots) planted perpendicular to rows of dry beans (main plots).

A field experiment was established under sprinkler irrigation at the University of Wyoming James C. Hageman Sustainable Agriculture Research and Extension Center near Lingle, Wyoming in 2009 and repeated in 2010 close to the same field (42°05'N, 104°23'W; altitude of 1,390 m above sea level). Soil at the site was a Haverson and McCook loam (42% sand, 41% silt, 17% clay, 1.9% organic matter, pH 7.9). The study was a two-factor factorial strip plot randomized complete block design with four replicates. The first factor consisted of dry bean market class and included 3 levels: ‘Schooner’ navy bean, ‘T-39’ black bean, and a control with no beans planted. The second factor consisted of grass species and included 5 levels: ‘Gulf’ annual ryegrass, ‘Oslo’ spring wheat, ‘Russell’ oat, ‘Pioneer 38N85’ corn, and a control with no grass planted. Grass species were planted perpendicular to dry bean rows, and levels of each factor were randomly assigned to plots within each replicate (Fig. 1). Plots were 3 by 4 meters. Dry bean and corn were planted in 76 cm row spacing using a John Deere Maxemerge 7300, 4-row, vacuum planter. In both the grass intercropped and monoculture bean plots, beans were seeded at a density of 163,000 seeds ha^−1^ and corn was planted at a density of 79,000 seeds ha^−1^. Grass species other than corn were seeded using a Tye double disk drill at 20 cm spacing. Annual ryegrass was seeded at the rate of 22 kg ha^−1^ [31] and wheat seeded at 44 kg ha^−1^ [32]. Trials were planted on June 19, 2009 and June 1, 2010.

Halosulfuron-methyl (‘Permit’ – Gowan Company) herbicide was applied pre-emergence over the entire trial area for weed control. Weeds surviving herbicide treatment were removed by hand as needed. Clethodim (‘Select’ – Valent U.S.A. Corporation) herbicide was applied to all intercropped plots 4 weeks after planting to selectively remove grass species.

Chlorophyll intensity in dry bean leaves was measured nondestructively using a SPAD-503Plus meter when dry bean had 2 to 4 trifoliolate leaves, and again at the 8 to 16 trifoliolate leaf stage. Two to three of the youngest fully expanded leaves from an average of 3 consecutive plants from the two middle rows of each plot were measured. Conversion of SPAD units to actual chlorophyll content requires calibration of SPAD chlorophyll meter readings using calibration curves developed from actual spectrophotometric measurements of extracted chlorophyll [33], [34], [35], [36]. We did not directly quantify chlorophyll content in this study. Previous studies have shown that SPAD readings correlate closely with direct spectrophotometric measurements of extracted chlorophyll [33], [34], [35], [36], [37], therefore, SPAD readings are presented as an approximation of chlorophyll intensity in bean tissue.

Dry bean leaf tissue samples were collected when dry bean had 2 to 4 trifoliolate leaves and again at the 8 to 16 trifoliolate leaf stage in both years. In 2010, additional bean leaf samples were collected at 4 to 8 trifoliolate leaf stages, and again at flowering. Two to three of the youngest fully expanded leaves were collected from an average of 30 consecutive plants from the two middle rows of each plot [38]. Tissue samples were rinsed in deionized water and dried at 60°C for 72 hours in accordance with Campbell and Plank [39] and then analyzed at Ward Laboratories, Inc. Kearney, Nebraska. Zinc (Zn), Fe, and Mn were determined using the Inductively Coupled Plasma Spectroscopy method [40]. Bean Tissue NO_3_-N was determined using the phenoldisulfonic acid (PDA) procedure described by Johnson and Ulrich [41] and detected using the flow injection analysis.

Soil samples were collected from each plot at planting and again when dry bean had 2 to 4 trifoliolate leaves. In 2010, additional soil samples were collected at 4 to 8 and 8 to 16 trifoliolate stage, and at bean flowering. Each soil sample consisted of five soil cores randomly located within each plot to 15-cm depth using a 2.5-cm diameter soil probe. Cores from each plot were thoroughly mixed together into a composite sample, dried at 60°C for 72 hours, and then shipped to Ward Laboratories, Inc. Kearney, Nebraska for analysis. Soil samples were analyzed for Fe, Zn, NO_3_-N, soil organic matter (SOM), pH, and Mn. Soil Fe, Zn, and Mn were extracted from soil samples using the DTPA micronutrient extraction method developed by Lindsay and Norvell [42]. Inductively coupled plasma atomic emission spectrometry [42], was used to determine the analytical concentration of the micronutrients.

The pH of the samples was determined using the saturation paste method [43] while SOM was determined using the ‘loss of weight on ignition’ method based on [44] and described by Combs and Nathan [45]. Soil NO_3_-N was extracted with 2 M potassium chloride in accordance with Keeney and Nelson [46].

At dry bean maturity, yields were measured by harvesting three meters of row from the middle two rows with a plot combine in September. A three meter pole was used to determine the length of rows from which to harvest; beans from those rows were pulled out and air dried in the field for five days before threshing and weighing using a plot combine. Yields were adjusted to 13% moisture content.

We reviewed the data and removed a single outlier in the 2009 pre-treatment soil NO_3_-N data [47]. Soil and tissue nutrient content (Fe, Zn, Mn, and NO_3_-N), soil pH, SOM, dry bean leaf chlorophyll intensity, and dry bean grain yield were subjected to analysis of variance (ANOVA) using the MIXED procedure of SAS [48]. The effect of grass species (none, oat, corn, annual ryegrass, or wheat), dry bean market class (Navy and black), and sampling date within a year were considered fixed effects in the model, while year and block within year were considered random effects. Treatment means were separated using Fisher's protected LSD (α = 0.05).

## Results

### Chlorophyll Intensity

**Figure 2:**
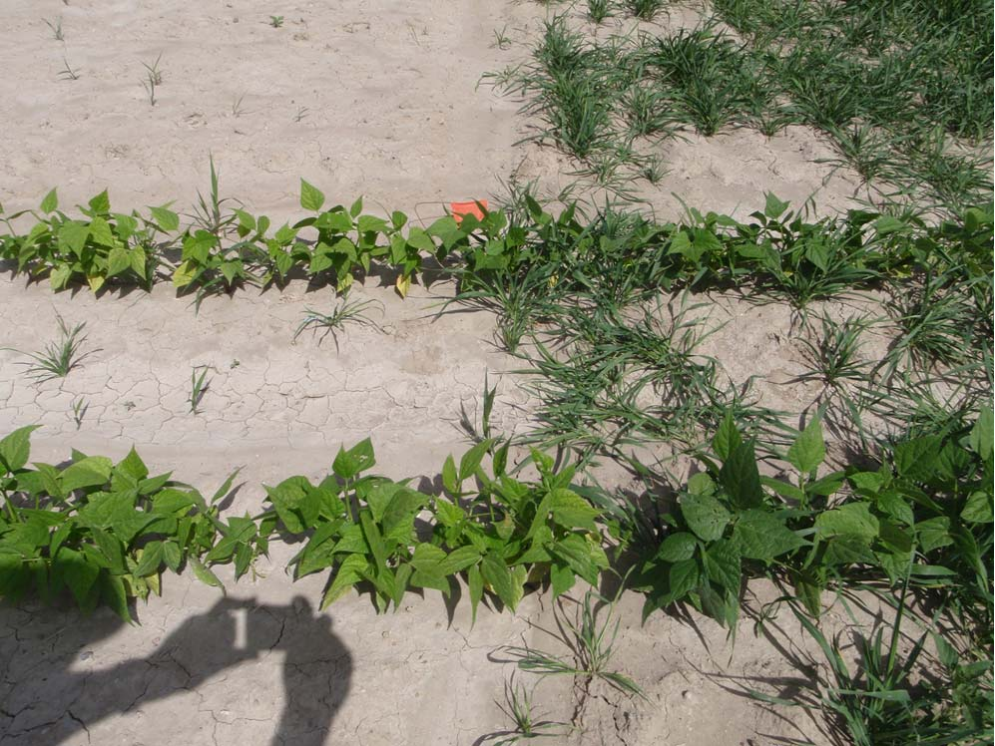
Picture taken in 2009 showing that beans planted in monoculture appeared more chlorotic than those in grass intercropped plots.

There was a significant (P<0.001) year by date by market class interaction for bean leaf chlorophyll intensity. The three-way interaction was due to black bean having a lower chlorophyll intensity compared to Navy bean at the second sampling date in 2010, but no differences among bean market class were observed at other sampling dates (data not shown). There was a significant date by grass interaction for chlorophyll intensity (P = 0.002), thus data was analyzed separately by sampling date. Chlorophyll intensity was greater in all grass-intercropped beans at 2 to 4 trifoliate leaf stage compared to bean monoculture (Fig. 2; Table 1). By the second sampling date, however, chlorophyll intensity had increased in the bean monoculture and decreased in the grass-intercropped treatments such that no differences between treatments were observed at the bean 8 to 16-trifoliolate stage (data not shown).

**Table 1:** Effect of grass species intercrops on bean leaf chlorophyll intensity (SPAD units), bean tissue and soil nutrients (mg kg^−1^) and dry bean grain yield (tons ha^−1^) in 2009 and 2010 near Lingle, WY.

Year		SPAD	Bean tissue Fe		Bean tissue Zn		Bean tissue Mn		Soil Zn	Soil NO_3_-N	Dry bean
		2-4TL	2-4TL	8-16TL	2-4TL	8-16TL	2-4TL	8-16TL	2-4TL	2-4TL	yield
2009	Bare soil	—	—	—	—	—	—	—	1.78ab	23.85ab	—
	Bean monoculture	29.68b†	814a	910a	29a	40a	115a	140a	1.81ab	29.20a	2.3a
	Bean + oat	37.20a	760ab	382c	22b	42a	97b	98c	1.67ab	17.85bc	1.5bc
	Bean + wheat	37.84a	739ab	353c	22b	40a	88b	81c	2.10a	15.94c	1.3c
	Bean + corn	36.89a	627b	620b	23b	42a	87b	105b	1.80ab	18.97bc	1.6bc
	Bean + ryegrass	36.30a	740ab	577 b	24b	39a	97b	97c	1.41b	23.64abc	1.7ab
									2TL to Flowering	2TL to Flowering	
2010	Bare soil	—	—	—	—	—	—	—	2.21	21.76ab	—
	Bean monoculture	25.40b	632‡	234	76	103	65	63	2.27	26.30a	2.8
	Bean + oat	33.68a	490	315	69	58	60	49	2.06	14.48c	2.8
	Bean + wheat	32.34a	591	241	84	57	70	46	1.99	13.81c	2.9
	Bean + corn	32.73a	645	239	57	61	61	45	2.09	17.29bc	2.5
	Bean + ryegrass	32.89a	600	280	74	59	69	48	2.16	19.58bc	3.0

† Means within a column and year followed by the same letter are not statistically different (alpha  = 0.05).

‡ Means without letters were not significantly different from other treatments within a year.

### Tissue Analysis

Tissue nutrient data were analyzed separately for each year because tissue sampling was conducted at different times in 2009 and 2010 (twice and five times respectively). In 2009, there was a significant (P<0.05) sampling date by grass species interaction with respect to bean tissue Fe and Mn concentration, and a marginally significant (P = 0.10) date by grass species interaction with respect to tissue Zn concentration, thus data for these nutrients were analysed separately at each sampling stage.

In 2009, dry bean monoculture had greater tissue Fe concentration compared to corn intercropped beans at the first sampling date, and greater tissue Fe concentration than all grass intercropped beans at the second sampling date (Table 1). Dry bean monoculture had greater Zn concentration compared to grass intercropped beans at the first sampling date, but no differences in tissue Zn were observed among intercrop treatments at the second sampling date. There was significant increase in tissue Zn concentration with time in all treatments. Manganese concentration in dry bean tissue was greater in dry bean monoculture compared with all grass intercropping treatments at both sampling dates.

In 2009, dry bean monoculture had nearly twice as much tissue NO_3_-N (3,609 mg kg^−1^) than beans intercropped with any grass species (1,884 mg kg^−1^) (P<0.0001). However, there were no differences among intercrop treatments for bean tissue NO_3_-N in 2010. In fact, contrary to 2009, there were no differences in tissue Fe, Zn, or Mn due to intercropping treatment.

### Soil Analysis

Given that soil was sampled at different times in 2009 and 2010, (twice and five times respectively), soil nutrient data were analyzed separately for each year. No differences between treatments were present for any soil variable at the time of bean planting (data not shown). There were no differences between treatments for soil pH, soil Fe, or SOM in either year.

In 2009, none of the grass intercropped treatments had soil Zn concentrations different from either the bare soil or dry bean monoculture treatment (Table 1). The wheat intercropping treatment reduced soil NO_3_-N compared to either dry bean monoculture or bare soil, while oat and corn intercropped treatments had lower soil NO_3_-N compared with the dry bean monoculture. The ryegrass intercropping treatment was not statistically different from any other treatment with respect to soil NO_3_-N. The wheat intercropped treatment was the only treatment to cause a statistically significant (P<0.05) decline in soil NO_3_-N concentrations between planting and the bean 8 trifoliolate stage. The bean monoculture treatment caused a statistically significant increase in soil NO_3_-N concentrations between planting and the 8 trifoliolate stage (data not shown).

In 2010, a similar trend in soil NO_3_-N was observed; all grass intercrops had lower (P = 0.0004) soil NO_3_-N compared to the bean monoculture, while bare soil was intermediary (Table 1).

### Dry Bean Yield

There was a significant year by grass species interaction for dry bean yield, thus data for bean yield were analyzed separately by year. In 2009, the bean monoculture had significantly greater yields than grass intercropped beans except for beans intercropped with ryegrass (Table 1). Ryegrass intercropped beans had significantly greater yields than wheat intercropped bean, but these yields were not statistically different from corn or oat intercropped beans. In 2010, no differences between intercropping treatments were observed.

## Discussion

Symptoms of Zn, Mn, and Fe deficiency can be similar in dry bean. Symptoms of Zn deficiency include interveinal chlorosis of leaves, shortening internodes and rosetting of terminal leaves. Minimum soil Zn concentration (DTPA) before Zn chlorosis symptoms can occur is 0.3 mg kg^−1^ [10]. Average soil Zn concentration from our study (2 mg kg^−1^) was far above the threshold found by Fageria and Stone [10] as capable of causing Zn deficiency chlorosis. The deficiency range of soil Fe concentration is 2.5–5 mg kg^−1^ [49]. Average soil Fe concentration, on the other hand, was 5 mg kg^−1^ in 2009 which was within the range found by Jacobsen et al. [49] as capable of causing Fe chlorosis. Although symptoms of Fe deficiency also include interveinal chlorosis of terminal leaves with the main veins remaining green [50], [51], rosetting of terminal leaves typical of Zn deficiency was absent in our study. Manganese deficiency can also result in interveinal chlorosis of leaves. However, average soil Mn concentration (DTPA) in our study was 7.35 mg kg^−1^, which was above the 6 mg kg^−1^ recommended by Fageria and Stone [10] as sufficient for dry bean production. These results therefore suggest that the chlorosis symptoms observed in the field study were caused by Fe deficiency rather than Zn or Mn deficiency.

High concentration of Zn, Mn, and several other heavy metals might compete with normal Fe uptake resulting in Fe deficiency chlorosis [52], [53], [54], [55], [56]. Both nutrient supply and nutrient balance are important considerations in plant nutrition as the concentration of one nutrient in the soil will often affect the uptake or transport of another nutrient within the plant [57]. However, while the soil Zn concentration (DTPA) in our study was above the threshold capable of causing Zn deficiency chlorosis [24], this concentration was still far below the toxic levels [24], [29] capable of competing with Fe uptake and is, therefore, unlikely to have had an impact on the performance of beans. Depressive effect of Mn on Fe uptake can occur when ferrous Fe is oxidized by Mn^4+^ non-enzymatically to ferric Fe [58] thus reducing Fe uptake, which is normally taken up as Fe^2+^ [56]. While results from our study showing significantly greater bean tissue Mn concentration (DTPA) in bean monoculture compared to grass intercropped beans in 2009 (Table 1), and a similar non-significant trend in 2010, suggest that Mn may have contributed to Fe deficiency chlorosis in bean monocultures, bean tissue concentration of Mn (averaging 127.5 mg kg^−1^ in 2009) was below the 400 mg kg^−1^ determined by Fageria and Stone [10] as sufficient for dry beans. The mean concentration of soil Mn concentration (DTPA) in monoculture bean plots (7.22 mg kg^−1^) was also only slightly above the 6 mg kg^−1^ determined by Fageria [59] and Fageria and Stone [10] as adequate for dry bean production. Also, no effect of tissue or soil Mn concentrations were observed in 2010. It is, therefore, unlikely that Mn had a substantial if any effect on Fe deficiency chlorosis in the dry beans.

Even though the dry bean monoculture exhibited greater Fe deficiency symptoms than intercropped beans, the monoculture had greater concentrations of Fe (DTPA) in leaf tissue. Several studies have shown that plants growing in high pH calcareous soils exhibiting Fe deficiency chlorosis frequently have comparable or higher Fe concentrations than green ones [60], [61]. This is explained by a phenomenon referred to as the "chlorosis paradox" described by Marschner [4], Abadia [62], and Morales et al. [60] attributed to the localization and binding state of Fe in leaves [4], whereby some of the Fe may precipitate in the apoplasm of leaves and become less available physiologically [4], [63]. Studies by Mengel [3] showed that Fe (III) reduction and Fe uptake from the apoplast into the cytosol in the leaves is affected by NO_3_ and ^–^HCO_3_ in the same way as in the roots. Transportation and reduction of Fe (III) to Fe (II) is mediated by a system of ferric-chelate reductase ensymes [61], whose activity is depressed at high pH [3]. Mengel [3] hypothesized that ^–^HCO_3_ in the root or leaf apoplast can neutralize H^+^ pumped out of the cytosol, which, together with NO_3_ taken up by H^+^/NO_3_ cotransport across the plasma membrane, can result in high pH levels at the root and leaf apoplast, thereby hampering or blocking Fe (III) reduction. The authors found a highly significant negative correlation between the leaf apoplast pH and the degree of Fe chlorosis in susceptible plants. These studies are consistent with our study findings that grass intercropped treatments tended to have less soil and tissue NO_3_-N, suggesting that high NO_3_-N in the monoculture treatments interfered with Fe metabolism in bean leaves, depressed chlorophyll synthesis, and induced Fe deficiency chlorosis.

Our results are also consistent with findings by Bloom et al. [20] that oat, planted as a competition crop just ahead of planting soybean, then killed with glyphosate herbicide at the height of 30 centimeters, absorbed NO_3_-N thereby reducing the amount available to the soybean crop and alleviated Fe deficiency chlorosis in soybean compared to soybean grown without the oat treatment. Our data suggest a similar finding. Lower soil NO_3_-N concentration in plots with grasses suggests that grasses reduced soil NO_3_-N concentration in those plots. Nitrogen fixation by Rhizobia associated with the legume may also have contributed to the greater NO_3_-N concentration in bean monoculture plots. Intercropping dry beans with annual ryegrass, spring wheat, oat, or corn as a means to reduce NO_3_-N in the soil and bean tissues may therefore provide a potential management tool for Fe deficiency chlorosis induced by high NO_3_-N in dry beans.

Although soil moisture was not measured in our study, grasses may also have reduced excess moisture from the soil that might induce Fe deficiency chlorosis. Studies have shown that increase in soil moisture can increase soil solution bicarbonate, which in turn can induce Fe deficiency chlorosis [20], [64). Inskeep and Bloom [65] found a highly significant correlation in soil moisture with Fe deficiency chlorosis in soybeans. However, irrigation was scheduled for bean monoculture, so it is unlikely that soil moisture played a major role in the Fe deficiency symptoms.

Soil splash may also have contributed to high dry bean tissue Fe concentrations measured across-the-board in our experiment [20], [51], [66]. A study by Inskeep and Bloom [51] measured Fe concentrations of 100 mg kg^−1^ and 70 mg kg^−1^ respectively in non-chlorotic and chlorotic soybeans leaves grown in Calciaquoll soils in pots. This contrasted with their findings in fields with similar soils that revealed soybean leaf tissue concentration ranging from 108 to 236 mg kg^−1^. They speculated that greater leaf Fe in chlorotic leaves is caused by fine soil particles containing aluminosilicates on both leaf surfaces that may not be completely removed despite careful washing of leaves in preparation for analysis [20]. Lower Fe concentration in grass intercropped bean tissues in our study may have been due to reduced raindrop and/or irrigation water impact on soil surface because of the increased plant cover. However, the grass species were still relatively small at the time of removal with the herbicide, so this protective effect was unlikely to have contributed much to treatment differences. Similar results were observed by Bloom et al. [20] who measured greater Fe concentration in monoculture soybean leaf tissues compared with soybean intercropped with oats. In a pot experiment, Inskeep and Bloom [65] measured greater Fe concentration in non-chlorotic compared to chlorotic soybeans leaves, but they did not find a significant correlation between leaf tissue Fe concentration and Fe deficiency chlorosis or soybean yields.

While results from our study showed that grasses used in this study can mitigate early season chlorosis symptoms in dry beans, grass intercropping did not result in greater dry bean yield. In fact, competition from the intercropped grasses (probably for water) decreased dry bean yield in 2009, although no negative effect on dry bean yield from grass intercrops was observed in 2010. Greater precipitation in 2010 (475 mm) compared to 2009 (404 mm) may have mitigated grass-bean competition for water. Greater yield in bean monoculture, despite observations and analytical results showing that they were more chlorotic than grass intercropped beans, suggest that benefits accruing from grass intercropping may have been outweighed by competition from grasses for nutrients, water, or light. Additional studies are required to determine the appropriate grass and dry bean densities, as well as the optimum time of removal of grasses from the intercropped treatment. Further research should focus on determining whether it is possible to find a grass removal timing that will both alleviate Fe deficiency chlorosis symptoms, but reduce the duration of competition so that a corresponding increase in dry bean yield is observed. This research will only be useful in a location where early season chlorosis symptoms are severe enough to significantly reduce dry bean yield.

## Conclusions

Our results showing that intercropping dry bean with annual grasses and other grass species can reduce Fe chlorosis symptoms early in the season are in agreement with Bloom et al. [20] who found similar results in soybean. Our results also suggest that annual ryegrass and oats may be better intercropping grasses compared to corn and wheat. This is because bean yields were less affected by annual ryegrass compared to wheat in 2009 and corn may be a more expensive crop in terms of seeds and fertilizer requirements and does not provide as good a ground cover as ryegrass and oat. Annual ryegrass' suitability is further enhanced by its slow and less prolific early season germination and establishment when soil and air temperatures are cooler [31], [67] and when Fe deficiency chlorosis is most severe. This, along with relatively lower stature of both grass species, makes them potentially less competitive for water and sunlight. Further research should investigate the potential of growing ryegrass and/or oat in the fall as winter cover crops and killing them the following spring as a means to reduce residual nitrates in the soil that can induce Fe deficiency chlorosis in subsequent dry bean or other susceptible crop.
